# Effectiveness of Gerontechnology Empowerment Program on Awareness and Use of Mobile Apps Among Older Adults for Instrumental Activities of Daily Living: Protocol for a Cluster Randomized Controlled Trial

**DOI:** 10.2196/53587

**Published:** 2024-05-13

**Authors:** Y N Shashidhara, G Raghavendra, Poornima P Kundapur, V Binil

**Affiliations:** 1 Manipal College of Nursing Manipal Academy of Higher Education Manipal India; 2 Welcomgroup Graduate School of Hotel Administration Manipal Academy of Higher Education Manipal India; 3 Manipal Institute of Technology Manipal Academy of Higher Education Manipal India

**Keywords:** gerontechnology, older adults, awareness, older people, instrumental activities of daily living, iADLs, mobile apps, mobile phone, empowerment

## Abstract

**Background:**

Instrumental activities of daily living (iADLs) are crucial for older adults to live independently. Health care and technological advancements will increase the older adult population and life expectancy globally. Difficulties with iADLs impact older adults’ quality of life. Mobile apps can assist older adults, but many require help due to limited awareness. Lack of awareness is a barrier to app use. Existing literature mainly covers health care and app design, needing more focus on iADL apps for older adults.

**Objective:**

The study objectives encompass 2 main aspects: first, to evaluate the awareness, use, and factors influencing the use of apps among older adults for iADLs; and second, to create and assess the effectiveness of a gerontechnology empowerment program (GEP) for older adults on the awareness and use of apps for iADLs.

**Methods:**

This research uses a quantitative approach divided into 2 distinct phases. In phase 1, we conduct a descriptive survey to assess the level of awareness and use of mobile apps for iADLs and identify the factors that influence the use of such apps among older adults. To ensure clarity and comprehension among participants, we provide them with a subject information sheet in both Kannada and English. The data collected during this phase enable us to gain insights into awareness levels, use patterns, and factors that shape older adults’ use of apps for iADLs. The results serve as the foundation for designing the GEP. In phase 2, a cluster randomization method will be used to select older adults aged 60 to 75 years in Udupi district, Karnataka, India, who are active smartphone users. These participants will be divided into 2 groups: the experimental and the control groups. The experimental group will join the GEP. The sample size for phase 1 is 554, and phase 2 is 50. To assess the effectiveness of this program, we will measure the outcomes before and after its implementation using the same assessment tools used in phase 1.

**Results:**

This study is funded by the Indian Council of Medical Research (Adhoc/193/2022/SBHSR on November 18, 2022). Phase 1 data collection is expected to be completed by November 2023, and phase 2 is scheduled to commence in the upcoming months. Phase 1 and 2 findings will be analyzed and discussed in the main paper, which we intend to submit to a high-quality peer-reviewed journal for publication. The research protocol, informed consent forms, and associated documentation received approval from institutional ethics committees (214/2020).

**Conclusions:**

Upon the successful testing of the GEP, it can be recommended that welfare departments encourage older adults to use mobile apps for iADLs and establish training programs to provide support to older adults in using these apps.

**Trial Registration:**

Clinical Trials Registry - India CTRI/2020/09/027977; https://ctri.nic.in/Clinicaltrials/pmaindet2.php?EncHid=NDUxMzM=&Enc=&userName=027977

**International Registered Report Identifier (IRRID):**

DERR1-10.2196/53587

## Introduction

### Background

Advancements in health care, lifestyle improvements, and technological progress are poised to elevate the global average life expectancy from 72.6 to 77.1 years by 2050, with India also experiencing a similar upward trajectory. In 1960, India had an overall life expectancy of merely 40 years, but today, it stands at approximately 69 years [1]. Forecasts indicate a continued increase in life expectancy in the coming decades [2]. According to a United Nations report, India’s older adult population is expected to grow substantially, reaching 19% by 2050 in contrast to the global average of 22%. Consequently, the aging demographic will encounter significant challenges in both activities of daily living (ADLs) and instrumental activities of daily living (iADLs), including tasks like communication, shopping, medication management, financial handling, travel, meal preparation, cleaning, and laundry [3]. This, in turn, will considerably impact older adults’ overall quality of life (QOL). Given these evolving trends, it becomes increasingly crucial for researchers and practitioners to identify cost-effective, feasible, and practical strategies to mitigate or prevent any decline in ADL and iADL abilities among older adults [4].

Experts consider the judicious use of technology as a facilitator and a potential solution for enhancing the QOL among the older population [5]. Specifically, mobile technologies and smartphones have proven highly effective in assisting older adults in self-management and prosperous living by providing them with increased access to relevant, valuable, and timely information for efficient self-governance, addressing ADLs and iADLs [6]. However, despite a growing inclination among older adults in India toward adopting mobile-related technologies, it is evident that they need help with mobile technology adoption [7]. Nevertheless, these challenges tend to diminish with the continuous use of mobile and related technologies, eventually becoming an integral part of their lives, aiding them in ADLs and iADLs [8]. Recognizing that smartphone technology use tends to be more prevalent among younger individuals, experts emphasize the importance of research that explores the motivations and barriers influencing smartphone and related technology use among older adults. This research is crucial because older adults stand to benefit as much, if not more, from technology compared to younger adults [9].

Considering the abovementioned observations, this research addresses these gaps by focusing on older adults. It aims to assess their awareness and use of mobile apps for iADLs, pinpoint the factors that drive or inhibit their use, and ultimately establish a sustainable gerontechnology empowerment program (GEP) based on the empirical findings [10]. This program aims to enhance and empower older adults’ propensity, interest, adoption, and use of the latest mobile apps designed for iADLs. This study is rooted in several interconnected areas, including the anticipated rise in the older adult population, the potential advantages of mobile apps in aiding iADLs, the factors influencing their adoption among older adults, and the empowerment of older adults in using mobile apps for iADLs. The demographic landscape of our world is undergoing a significant transformation, with older adults playing an increasingly substantial role. Older adults account for approximately 11.5% of the global population, projected to surge to 22% by 2050 [3]. The importance of iADLs in the lives of older adults cannot be overstated. These activities are notably more intricate than the basic ADLs and are pivotal for older adults to maintain their independence [11].

### Significance of the Study

In the context of ADLs, it is noteworthy that, as of 2011, around 7.6% of older adults in India faced difficulties in performing them. However, the distinction between ADLs and iADLs becomes more evident when we consider that iADLs demand heightened expertise, decision-making abilities, skills, and individual discretion compared to the tasks associated with ADLs [1]. The challenges older adults encounter in managing iADLs are well-documented by research, consistently affirming the previously mentioned perspective [11]. Studies have shown that older individuals experience escalating difficulties in food preparation, shopping, handling telephones and related devices, and basic laundry [12]. These challenges underscore the pressing need for research and intervention to facilitate the adoption of technology, particularly mobile apps, to empower and support older adults in their pursuit of independent living [7]. Older adults’ use of technology, including computers and the internet, is influenced by various factors, including age, education, and attitudes toward technology [13]. Previous research has consistently shown that age is a strong negative predictor of technology use, suggesting that older individuals tend to be less inclined to embrace technological advancements [14].

In the context of developing countries, mobile phones, mobile apps, and related technologies have emerged as crucial components of information and communication technology (ICT) systems designed to assist individuals of all ages [15]. These technologies have found significant use, particularly in the health care sector. Mobile apps, as integral components of mobile health (mHealth) interventions, offer a wide range of benefits, such as assessing fall risks in home settings for older adults [14]. Mobile phones also play a vital role in supporting telemedicine and providing remote health care services in developing nations [16].

The advent of net banking has brought about advantages for older adults, including 24-hour access, flexibility, independence, and the ability to overcome physical barriers associated with age in accessing financial services. Similarly, mobile apps related to food preparation, such as recipes and diet apps, as well as service-oriented apps for housekeeping, laundry, and cleaning, have the potential to enhance various aspects of older adults’ lives. However, despite the widespread adoption of mobile phones and related technologies and their proven potential to improve services like health care, banking, and travel, these technologies’ adoption, acceptance, and use among older adults remain notably low [17].

Mobile phones and related technologies and apps have demonstrated their capacity to significantly enhance the QOL for older adults in areas such as health care, commerce, independent living, reduced isolation, strengthened connections with family and friends, and more [9,18].

Connecting with loved ones through technology contributes to higher mental and physical well-being. Notably, technology use for nonsocial purposes is linked to enhance the overall physical well-being, while its social use mitigates the negative impact of loneliness on overall life satisfaction [19]. Consequently, mobile phones and related technologies and apps extend beyond mere communication devices; they have a profound societal impact by liberating us from geographical constraints.

Despite older adults demonstrating a generally positive attitude toward technology, they often encounter complexities. Additionally, demographic characteristics such as age, gender, and education level play crucial roles in predicting their adoption of mobile technology [20]. Understanding the motivations and obstacles behind older adults’ behavior regarding the use of mobile apps is essential [9].

In the context of mHealth adoption among older adults in a developing country, a study applied parameters from the well-established unified theory of acceptance and use of technology framework along with 2 additional variables: technology anxiety and resistance to change. The study revealed that factors such as performance expectancy, social influence, technology anxiety, effort expectancy, and resistance to change significantly influenced older adults’ behavioral intentions to use mHealth. Interestingly, facilitating conditions did not substantially affect behavioral purposes [17]. This underscores the importance of considering the perceived benefits and ease of use and factors like anxiety and resistance to change when designing interventions to promote technology adoption among older adults.

A randomized controlled trial was implemented on smartphone use training programs among older adults and found a positive effect on smartphone use skills and overall QOL. This underscores the importance of providing detailed instructions and guidance to older adults by technology and service providers to cultivate favorable perceptions and confidence in using new technology [10]. Furthermore, any training program designed for older adults to promote the practical use of mobile apps is expected to result in significantly lower depressive symptom scores than baseline measurements. Additionally, it is anticipated that such programs will facilitate the use of mobile apps for various purposes, including health management, entertainment, transportation, and social media interaction, thereby contributing to the overall well-being of older adults [21]. While the impact of ICT on ADLs has been acknowledged, more research needs to examine the influence of ICT on iADLs [22]. Given the increasing older adult population, there is a pressing need for a comprehensive understanding of effective empowerment and training interventions tailored to their unique needs [23]. This will be instrumental in improving their QOL and enabling them to lead more independent and fulfilling lives. With this study, we would like to contribute to achieving the Sustainable Development Goal (4.3.2) by educating the general public and encouraging lifelong learning.

### Novelty

This study intends to fill the following gaps in the literature: (1) While there is substantial literature on mobile technology and apps for older adults in the context of their iADLs, existing studies predominantly emphasize health care, app features, design considerations, and overall effectiveness. (2) This study uniquely focuses on using mobile apps by older adults to facilitate their performance of iADLs. (3) The GEP represents a pivotal aspect of this research, aiming to foster awareness and encourage the use of mobile apps among older adults. (4) This program empowers older adults to effectively perform their iADLs through technology.

### Objectives

The objectives of the study are to assess the awareness and factors influencing the use of mobile apps among older adults for iADLs and to develop and determine the effectiveness of a GEP for older adults on awareness and use of mobile apps for iADLs.

## Methods

### Research Design

The research design for phase 1 is cross-sectional descriptive research, and phase 2 is a cluster randomized control trial. The Indian Council of Medical Research Guidelines for Extramural Research was adopted to prepare the funding proposal [24].

### Participants

The participants in this phase exclusively consist of older adults aged between 60 and 75 years who use smartphones and are affiliated with welfare organizations associated with the District Disabled Welfare Office in Udupi district, Karnataka state, India. Additionally, participants must be able to read and write in English and express a willingness to participate in the study. Nonsmartphone users will be excluded from this research study.

### Sample Size

Sample size calculations were performed separately for phase 1 and phase 2 of the study. For phase 1, a sample size of 369 participants was estimated based on proportion values. This sample size was then adjusted by a design effect of 1.5 to account for potential clustering or stratification, resulting in a required sample size of 554 participants. In phase 2, the focus was on comparing 2 proportions. To ensure the study’s robustness in the face of potential dropouts (estimated at 20%), an initial sample size of 31 participants was calculated for each group. This initial size was adjusted by a design effect of 1.5 to consider the study’s specific design characteristics, yielding a total required sample size of 46 participants per group, rounded up to 50 participants per group.

### Ethical Considerations

The research protocol, informed consent forms, and associated documentation received approval from the institutional ethics committee (214/2020) of Kasturba Medical College and Kasturba Hospital Institutional (Registration no.: ECR/146/Inst/KA/2013/RR-19). This study is also registered under the Clinical Trials Registry - India (CTRI/2020/09/027977). In this study, participants will receive a subject information sheet in both Kannada and English to help them understand the study. The data will be collected only after informed consent is obtained. The confidentiality and anonymity of the participants will be maintained.

### Recruitment

**Figure 1:**
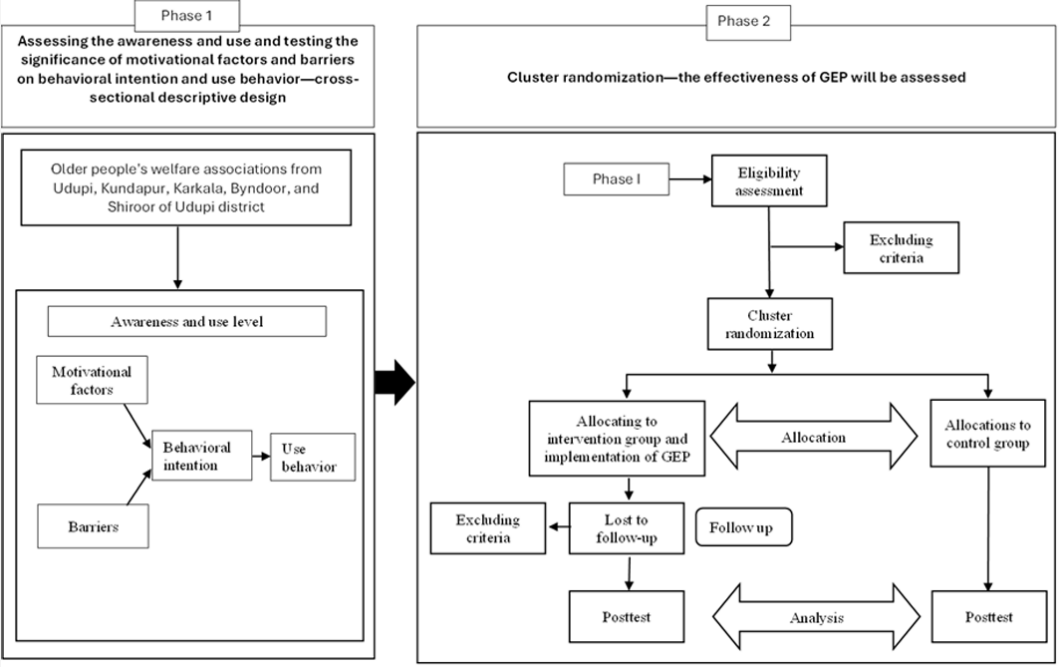
Research framework. GEP: gerontechnology empowerment program.

This study is structured around a 2-phase research design (Figure 1). The sample was selected purposively from welfare associations of the Udupi district. Phase 1 was initiated after the ethics and Clinical Trials Registry - India approval. For the study’s second phase, the sample will be randomly allocated to experimental and control groups from various welfare organizations of the Udupi district. We have planned to use the clustered randomization process to select participants based on the study’s eligibility criteria.

### Research Instrument

The data collection process for this study will involve the use of structured questionnaires in both English and Kannada. The questionnaire will cover the following key areas: demographic information—participants will provide essential demographic details, including age, gender, educational qualifications, income range, the presence of chronic diseases, and the type of smartphone they use; awareness and use level of mobile apps for iADLs—this section will measure older adults’ awareness and usage level regarding various mobile apps designed to address the 8 components of iADL; and motivational factors and barriers—this portion of the questionnaire will explore the motivational factors and barriers influencing older adults’ adoption and use of mobile apps. It will encompass dimensions such as performance expectancy, social influence, facilitating condition, effort expectancy, technology anxiety, awareness factors, and resistance to change.

Collecting data in both English and Kannada ensures that participants can comfortably respond in their preferred language, facilitating accurate and comprehensive data collection from a diverse group of older adults.

### Instrument Validity and Reliability

The instruments used in this study underwent a rigorous validation process. Experts from various fields, including health care, social studies, information technology, and the older adults’ welfare department, were engaged in the validation process. These experts were presented with a clear understanding of the study’s objectives and its blueprint. The reliability was established among 20 participants and evaluated through the test-retest method, ensuring consistent responses. Additionally, Cronbach α, a statistical measure of internal consistency, was used to assess the reliability of awareness and usage level on mobile apps for iADLs (*r*=88) and motivational factors and barriers (*r*=73) instruments. Furthermore, a pilot study was conducted among 10 participants to assess the study’s feasibility and accuracy in January 2023.

### Data Collection Plan

The study is in progress by visiting older adults’ welfare organizations after obtaining prior approval. In phase 1, participants will receive a subject information sheet in both Kannada and English to explain the study details. Data will be collected using a structured questionnaire, with participants’ consent, to assess their awareness and the factors influencing the use of mobile apps for iADLs. In phase 2, cluster randomization will be used to select older adults for the experimental or control groups. The GEP will be administered to the experimental group. The effectiveness of the GEP will be assessed through measurements taken before and after its implementation.

### Statistical Analysis

#### Plan for Data Analysis

To evaluate the awareness and use of mobile apps among older adults, frequency and percentage distribution will be used as a statistical method. A combination of statistical techniques will be used to analyze the influencing factors, including descriptive statistics to provide a summary of the data and logistic regression to model the relationship between the influencing factors and mobile app use. A sequence of statistical tests will be applied to measure the effectiveness of the GEP. These tests include repeated measures ANOVA, 2-tailed *t* test, Mann-Whitney *U* test, Wilcoxon signed rank test, and *χ*^2^ test. These tests will help assess the impact and changes brought about by the GEP on various outcome measures.

#### Develop and Determine the Effectiveness of a GEP

The GEP will be a comprehensive intervention designed to facilitate the use of smartphone apps for iADLs. The development of GEP will be based on the findings from the study’s first phase, incorporating insights from experts in health care, mobile technology, and older adults’ welfare departments or organizations. The structure of the GEP program will encompass the following components: information and features—detailed information about smartphone apps relevant to iADLs, including their features and functionalities; installation procedures—step-by-step guidance on installing and setting up these smartphone apps; general awareness—fundamentals of smartphone use and general awareness regarding its capabilities; security awareness—guidelines on ensuring smartphone security and safe use; dos and don’ts—best practices and precautions for effective and responsible smartphone use; operation manuals—manuals provide instructions on operating specific smartphone apps for iADLs. The training program will be conducted through 8 sessions, each lasting 3 hours. These sessions will incorporate hands-on training using audiovisual facilities to enhance participants’ understanding and practical skills in using smartphone apps effectively for iADLs.

## Results

### Overview

This study is funded by the Indian Council of Medical Research (Adhoc/193/2022/SBHSR on November 18, 2022). Phase 1 data collection is expected to be completed by November 2023, and phase 2 is scheduled to commence in the upcoming months. Phase 1 and 2 findings will be analyzed and discussed in the main paper, which we intend to submit to a high-quality peer-reviewed journal for publication. The research protocol, informed consent forms, and associated documentation received approval from institutional ethics committees (214/2020).

### Expected Outcomes

The expected outcomes are as follows: The data collected from this study will provide valuable insights into the actual awareness levels and the influencing factors affecting the use of mobile apps for iADLs among older adults. The design and testing of the GEP have the potential to significantly enhance the ability of older adults to manage iADLs, leading to improved self-management and overall well-being, which in turn has a positive societal impact. To further extend the benefits of this research, collaboration with nongovernmental organizations and self-help groups can be explored to implement the GEP on a larger scale. This would allow for a broader reach and greater empowerment of older adults. Additionally, the results of this study can serve as a valuable reference for other researchers seeking to empower older adults in using mobile technologies. As part of the GEP, developing the iADLs Guide, a mobile app providing regular updates on technology, will be an ongoing service for older adults. This tool will ensure older adults have access to the latest information and updates in the ever-evolving field of mobile technology, further enhancing their ability to perform iADLs effectively. This initiative contributes to achieving sustainable development goal 4.3.2 to provide education to the general public and encourage lifelong learning [25].

## Discussion

### Principal Findings

The primary objective of the GEP is to enhance awareness and use of mobile apps among older adults for iADLs. The study findings can improve older adults’ participation in the GEP, significantly increasing awareness and usage levels. This aligns with previous research, such as the study by Xie and Kalun Or [26], which demonstrated that targeted interventions like mHealth can effectively improve technology adoption and use among older adults. In our research, the GEP provided structured guidance, hands-on training, and resources to bridge the gap in awareness and facilitate the use of mobile apps for iADLs. One of the noteworthy outcomes of the GEP was its positive impact on the QOL among older adults. The program can empower older adults to perform iADLs more independently and efficiently, enhancing well-being. This finding resonates with existing literature. For instance, a study by Sen et al [27] emphasized through a systematic review that using technology, including mobile apps, can reduce social isolation, increase engagement in daily activities, and improve mental and physical well-being among older adults. The GEP’s focus on addressing the specific needs of older adults in iADLs aligns with the principles of person-centered care and empowerment.

The success of the GEP can be attributed to its tailored approach. The program was developed based on older adults’ specific needs and preferences, considering technological anxiety and resistance to change. This personalized approach aligns with the recommendations of Charness and Boot [28], who emphasized the importance of designing technology interventions that consider individual differences and provide ongoing support. The GEP’s combination of information, hands-on training, and ongoing assistance through the iADLs Guide mobile app ensured that older adults received the necessary support at every stage of their technology adoption journey. Collaboration with older adults’ welfare organizations, nongovernmental organizations, and self-help groups is pivotal in successfully implementing the GEP. This collaborative approach resonates with Baker et al [29] findings, highlighting the importance of involving community organizations in technology interventions for older adults. The positive outcomes observed in this study suggest that the GEP model can be scaled up and replicated in other regions and populations, further extending its benefits to a larger demographic of older adults.

This protocol addresses a pressing issue—the growing digital divide among older adults. Older adults may face challenges in keeping pace with technological advancements as the world becomes increasingly digital. This protocol aligns with the findings of the Pew Research Center, which reported that while technology adoption among older adults is rising, a substantial digital divide persists. The GEP represents an effort to bridge this divide and empower older adults to leverage technology for iADLs. This protocol builds on prior research, highlighting the potential benefits of technological interventions for older adults. For example, a systematic review by Wildenbos et al [30] emphasized that technology interventions can enhance older adults’ independence, well-being, and social connectivity. This protocol seeks to contribute to the existing body of knowledge by assessing the effectiveness of a structured empowerment program tailored to older adults’ unique needs and challenges in adopting mobile apps for iADLs.

The study plan and methodology emphasize on assessing the real-world impact of the GEP. While many studies have explored the potential benefits of technology for older adults in controlled settings, this protocol seeks to evaluate the program’s effectiveness in the context of participants’ daily lives. This aligns with the recommendations of Mitzner et al [31], who stressed the importance of studying technology use in ecologically valid environments to capture its true impact. Therefore, this protocol for the effectiveness of GEP represents a significant endeavor to empower older adults with the necessary skills and knowledge to use mobile apps for iADLs. Drawing from supportive studies and available evidence, the protocol underscores the importance of addressing the digital divide, tailoring interventions to individual needs, and collaborating with stakeholders to enhance the well-being and independence of older adults in the digital age.

### Limitations

Acknowledging the study protocol’s limitations and ethical considerations is crucial. The sample size may be restricted to a specific geographic area, and participants will need a baseline level of English literacy. Researchers must ensure informed consent and safeguard participants’ privacy and data security, following established ethical guidelines.

### Conclusions

Following the successful testing of the GEP, there will be an opportunity to recommend its implementation to welfare departments. This proposal encourages older adults to embrace mobile apps for performing iADLs. Additionally, establishing a mobile app training initiative can provide training and support to older adults in using these apps effectively.

## Abbreviations

ADL: activity of daily living
GEP: gerontechnology empowerment program
iADL: instrumental activity of daily living
ICT: information and communication technology
mHealth: mobile health
QOL: quality of life
